# Urbanization Altered Bacterial and Archaeal Composition in Tidal Freshwater Wetlands Near Washington DC, USA, and Buenos Aires, Argentina

**DOI:** 10.3390/microorganisms7030072

**Published:** 2019-03-06

**Authors:** Martina Gonzalez Mateu, Cedric Evan Park, Cullen Patrick McAskill, Andrew H. Baldwin, Stephanie A. Yarwood

**Affiliations:** Environmental Science and Technology Department, University of Maryland, College Park, MD 20742, USA; gmateu@umd.edu (M.G.M.); cpark126@umd.edu (C.E.P.); mcaskill@terpmail.umd.edu (C.P.M.); baldwin@umd.edu (A.H.B.)

**Keywords:** urbanization, tidal freshwater wetlands, soil microbial communities, 16S rRNA, xenobiotic degradation, nitrification, methanogens

## Abstract

Urban expansion causes coastal wetland loss, and environmental stressors associated with development can lead to wetland degradation and loss of ecosystem services. This study investigated the effect of urbanization on prokaryotic community composition in tidal freshwater wetlands. Sites in an urban, suburban, and rural setting were located near Buenos Aires, Argentina, and Washington D.C., USA. We sampled soil associated with two pairs of functionally similar plant species, and used Illumina sequencing of the 16S rRNA gene to examine changes in prokaryotic communities. Urban stressors included raw sewage inputs, nutrient pollution, and polycyclic aromatic hydrocarbons. Prokaryotic communities changed along the gradient (nested PerMANOVA, Buenos Aires: *p* = 0.005; Washington D.C.: *p* = 0.001), but did not differ between plant species within sites. Indicator taxa included *Methanobacteria* in rural sites, and nitrifying bacteria in urban sites, and we observed a decrease in methanogens and an increase in ammonia-oxidizers from rural to urban sites. Functional profiles in the Buenos Aires communities showed higher abundance of pathways related to nitrification and xenobiotic degradation in the urban site. These results suggest that changes in prokaryotic taxa across the gradient were due to surrounding stressors, and communities in urban and rural wetlands are likely carrying out different functions.

## 1. Introduction

Global urbanization has rapidly increased throughout the last 60 years, and by 2050, two-thirds of the world population will live in urban settlements [1]. Population growth and the resulting urban expansion is a major cause of wetland loss worldwide [2,3]. Coastal wetlands are particularly susceptible to loss and habitat degradation, as the most dramatic population growth has occurred in cities located near coastlines [4,5]. 

Urban development has a direct effect on wetland ecosystem services, and the loss and degradation of these systems can impact human health and well-being [6]. Increased impervious surfaces facilitate the transport of pollutants into waterways and directly impact wetland habitat quality [7]. On average, in the United States, urban storm water runoff can carry 0.26 mg/L of phosphorus, 0.2 mg/L of nitrogen, and 54.5 mg/L of suspended sediments [8], which can transport various contaminants like heavy metals and polycyclic aromatic hydrocarbons (PAHs) [9,10]. As a result, urban wetlands tend to have more non-native plant species, typically favored by nutrient inputs, and higher sediment concentrations of heavy metals and organic chemicals [11,12]. 

Changes in urban wetland habitat can affect the diversity and richness of biota. For example, urbanization has been seen to reduce macro-organism species diversity by creating homogeneous plant and animal assemblages [13,14]. Johnson et al. [15] reported that urban wetlands had significantly less richness of aquatic insects, mollusks, amphibians, aquatic reptiles, and crayfish than wetlands in non-urban systems. A study on urban rivers, which are subject to similar stressors as urban wetlands, found microbial richness decreased in the urban areas and had higher abundance of genes related to xenobiotic metabolism [16]. To our knowledge, no studies have looked at the effects of urbanization on tidal freshwater wetlands (TFW) prokaryotic communities, but it is expected that they would also be susceptible to anthropogenic disturbances [17,18].

Wetland prokaryotic communities may change directly in response to added nutrients or shifts in the length and intensity of the hydroperiod, but may also indirectly be impacted by vegetation. In order to investigate the role of different plant functional groups on wetland prokaryotic communities, we sampled the root zones of two plant species. Root morphology and plant type can influence rhizosphere prokaryotic communities through plant exudates and oxygen release [19,20]. Some studies have found that prokaryotic community structure can vary between wetland plant species [21] and even between lineages of the same species [22,23]. Other studies reported site-specific effects [24] or found that soil characteristics were the main drivers of microbial community composition [18]. 

We examined the prokaryotic communities in TFWs located near two major capital cities: Buenos Aires (Argentina), and Washington, D.C. (United States). Tidal freshwater wetlands are located in the upper part of estuaries and are characterized by salinity levels lower than 0.5% [25], and daily tidal fluctuations due to the influence of the nearby estuary. Their location often represents the most inland point that can still be reached by ships in the estuary, supporting early settlements in these tidal areas that continued to develop over the next few centuries [26]. As a result of increased economic activity and population growth, tidal wetlands are particularly vulnerable to environmental degradation and loss associated with urban development [27,28]. 

Our study used next-generation DNA sequencing to test three hypotheses: (1) Prokaryotic community composition and function will vary across the established urban gradient; (2) prokaryotic communities will differ between plant species at each site; and (3) specific prokaryotic taxa will be indicative of urban wetlands, regardless of geographic location of the city.

## 2. Materials and Methods 

### 2.1. Site Description

The cities of Buenos Aires and Washington D.C. are two examples of large cities that developed near tidal freshwater wetlands. Both cities are located at similar latitudes North and South of the Equator (Buenos Aires: 34.6037° S; D.C.: 38.9072° N) and have experienced significant population growth over the last few decades. This trend is expected to continue in both areas, and current population estimates are around 3 million people in Buenos Aires with ~376,000 residents in the county where we sampled [29], and almost 700,000 in Washington D.C. [30].

Argentinian sites were located in the Lower Paraná Delta, in the upper portion of Rio de la Plata estuary. Islands formed in this area in the last ~750 years and the delta continues to expand due to sediment deposition [31]. About half of this area has been affected by human activities, mainly forestry and development associated with tourism and recreation [32]. To allow these activities, hydrological modifications like levees and dikes were constructed, which altered the natural environment and affected hydrologic regimes [33]. Wetlands in the Lower Paraná delta have been increasingly lost to urban expansion [34]. Most of the developed area uses septic systems, and as drainfields become less effective over time, wastewater is released into the waterways. Water quality in this area is also affected by inadequate disposal of solid wastes, industrial pollution, and fuel spills from heavy boat traffic [35,36]. 

The United States sites were located in the Chesapeake Bay estuary. These tidal marshes originated during the Holocene (~10,000 years ago), when Pleistocene valleys were gradually inundated by rising sea level [37]. As a result of human intervention and sea level rise, over half of the tidal wetlands in the Bay are considered to be degraded [38]. The main perturbations associated with development near these wetlands are pollutant runoff that is promoted by large impervious surfaces [27,39] and nutrient enrichment from agriculture and sewage treatment facilities [40]. Washington D.C. has a combined sewer overflow system, so after heavy rainfalls a mix of storm water and sewage is released into nearby waterways, introducing bacterial pathogens and degrading water quality [41].

**Figure 1:**
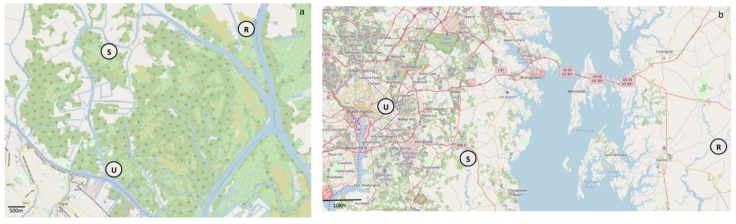
Location of sampling sites in Buenos Aires (**a**) and Washington D.C. (**b**). U = urban, S = suburban, and R = Rural. Base map: OpenStreetMap (https://www.openstreetmap.org).

The study sites are located in freshwater tidal wetlands in the Paraná River delta (Buenos Aires, Argentina), and in the Chesapeake Bay (Maryland, USA). Within each region, sites corresponding to urban, suburban, and rural environments were identified (Figure 1).

In Buenos Aires the sites were located in the Lower Paraná Delta, just north of the City of Buenos Aires. The urban gradient was established by locating sites that had varying degrees of development. The urban site is on the Sarmiento River near its confluence with the Luján River (34°24’48.81”S, 58°34’1.76”W), which is considered to be highly contaminated with wastewater and industrial waste [42]. The selected area is influenced by tidal inputs from the Luján River, experiences heavy boat traffic, and has been modified to accommodate residential houses. The suburban site (34°23’8.27”S, 58°34’6.30”W) is located upriver from the urban site, and the rural site is located on the Unión River (34°22’55.73”S, 58°31’38.77”W) on unmanaged land with no signs of human development upstream. 

In Maryland the sites are located in the Anacostia River (38°92′41.1′’N, 76°94′58.8′’W; soil series Zekiah and Issue), Patuxent River (38°78’58′’N, 76°71′30.8′’W; soil series Nanticoke and Mannington), and Choptank River (38°48’52.67”N, 75°53’19.82”W; soil series Nanticoke and Mannington). The Anacostia River runs along the border of Washington D.C. and is highly urbanized and affected by industrial activities and sewage inputs from the city’s combined sewer system. The Patuxent watershed is located between Washington D.C. and Baltimore, representing a site of intermediate urban development, and the Choptank River is located across the Chesapeake Bay in Eastern Maryland, where agriculture is the predominant land use [43].

We selected two species that have different morphological features at each site. In Washington D.C. the selected species were *Phragmites australis* (Cav.) Trin. ex Steud., and *Peltandra virginica* (L.) Schott, and in Buenos Aires, *Hymenachne grumosa* and *Sagittaria montevidensis*. *Phragmites* and *Hymenachne* are clonal grasses that have thick rhizomes, tall and rigid stems, and horizontal cable-like stolons. *Peltandra* and *Sagittaria* have fleshy triangular leaves and a shallower root system with bulbous vertical corms or tubers. In addition, *Phragmites* is an invasive species that has higher nutrient requirements [44] and produces more biomass than other native species [45].

### 2.2. Sample Collection

Samples were collected on a summer day at each location (January in Buenos Aires and August in Maryland). In Maryland, three soil samples were collected from the rhizosphere of *Phragmites australis*, and three from *Peltandra virginica* at each of the sites. We used a half circle Russian peat borer (Eijelkamp, Giesbeek, Netherlands) to collect a 50-cm deep soil sample next to the stem to get plant-influenced soil. In Buenos Aires four soil samples were collected from the rhizosphere of *Hymenachne grumosa* and *Sagittaria montevidensis*. We used a spade shovel with measurement markings to collect soils from a depth of 30 cm, as these plants have a shallower root system than those sampled near D.C. Approximately 2 g of homogenized rhizosphere soil were added into sterile Falcon tubes that contained 4 mL of LifeGuard soil preservation solution (MoBio Laboratories, Carlsbad, CA) and shipped to the University of Maryland for soil and prokaryotic community analysis.

### 2.3. Soil Analysis

Key soil properties known to have a significant impact on prokaryotic ecology were analyzed using the methods outlined by Yarwood et al. [22]. Total C and N content were determined by combustion analysis at 950°C and soil organic matter was calculated using loss-on-ignition (550 °C for 24 hr). To measure pH, five grams of soil were added to 25 mL of distilled water to make a 1:5 ratio slurry, which was then measured using a pH electrode. Finally, particle size analysis (PSA) was carried out using the hydrometer method for the Washington D.C. samples and the pipette method [46] for the Buenos Aires samples. Logistics of working in different countries prevented the use of the same core type and particle size analysis method.

### 2.4. DNA Extraction and Illumina Library Preparation

DNA extractions were carried out using the Qiagen DNeasy PowerLyzer PowerSoil kit (Qiagen, Hilden, Germany). The Buenos Aires samples were centrifuged, and the excess solution drained before beginning the extractions. These were carried out following the manufacturer’s instructions, except for the homogenization step that was done using a FastPrep-24 (45 s at 6 m/s; MP Biomedicals, LLC, Solon, OH). Samples were quantified using a Qubit 2.0 fluorometer (Invitrogen) and diluted to 5ng/ul for PCR amplification and subsequent amplicon sequencing. The 16S rRNA region was targeted using the primers 515F+adapter (5′-TCGTCGGCAGCGTCAGATGTGTATAAGAGACAGGTGCCAGCMGCCGCGGTAA-3′) and 806R+adapter (5′-GTCTCGTGGGCTCGGAGATGTGTATAAGAGACAGGGACTACVSGGGTATCTAAT -3′) [47]. The PCR reaction had 3.5 uL of DNA, 17.5 uL of ThermoScientific ^TM^ Phusion^TM^ Flash High-Fidelity PCR Mastermix (Thermo Fisher Scientific), and 7 uL of each primer (1 ng/uL). The PCR product was then processed for Illumina sequencing using the 16S Metagenomic Sequencing Library Preparation protocol (Part # 15044223 Rev. B, support.illumina.com). The cleanup was carried out using AMPure XP beads (Beckman Coulter, Pasadena, CA), and the Nextera XT 96 index kit (Illumina) was used for sample indexing. Samples were pooled, and amplicon size of the library was checked using a Bioanalyzer 2100 (Agilent Technologies). Q-PCR was used for library quantification, and the final library was diluted to 12 pM, spiked with 30% PhiX (Illumina), and run on an Illumina MiSeq using a 600-cycle v3 cartridge. 

### 2.5. Data Analysis

R was used for statistical analysis and drawing figures [48]. Illumina sequencing output was processed using the dada2 package (version 1.6) [49] for filtering, dereplication, sample inference, merging of pair end reads, and chimera checking. The algorithm used for chimera checking in this performs a Needleman-Wunsch global alignment of each sequence to compare it with more abundant sequences, and check if the “child” sequence can be obtained from exact combinations of right and left segments of “parent “sequences, which would classify them as chimeras. Taxonomic assignments were carried out by matching sequences to the SILVA database (SILVA v128, arb-silva.de) and the resulting amplicon sequence variant table was analyzed using the phyloseq (v1.2) [50] and vegan (v2.4-4) [51] R packages. Samples were rarefied to the minimum sequence number for each data set (Buenos Aires = 11,811, Maryland = 59,939). Rarefaction curves were produced and confirmed that coverage of sampling was appropriate, as all but one sample curve leveled off at the proposed sequence cut-off (Figure S1 in supplementary materials). Non-metric multidimensional scaling (NMDS) based on a Bray-Curtis dissimilarity matrix was used to visualize differences between sites and plant species, and homogeneity of group dispersion was checked using the vegan functions betadisper and permutest. Nested PERMANOVA was used to test for statistical differences with the adonis function and a calculated p value < 0.05 was considered significant. Significant factors identified through permutational multivariate analysis of variance (PERMANOVA) were further examined with pairwise comparisons using permutation MANOVAs to assess differences between group levels. This was done using the pairwise.perm.manova function from the RVaideMemoire package [52] with 999 permutations on the Bray-Curtis distance matrix and Bonferroni corrections to adjust p-values after multiple testing. The associations between community composition and the most abundant phyla were evaluated using the envfit function, and vectors that showed significant correlations were fitted to the non-metric multidimensional scaling (NMDS) ordination. Correlations between community composition and soil variables were also evaluated with the envfit function to identify significant environmental factors. Prokaryotic amplicon sequence variants (ASVs) were classified into urban or rural habitat generalist or specialist using a multinomial species classification method (CLAMtest) in vegan. We used a coverage limit of 10, an alpha of 0.005, and a specialization value of 0.67, which is considered conservative [53]. Furthermore, indicator taxa for urban and rural habitats were identified using the multipatt function in the indispecies package [54], and significant associations between taxa and sites were evaluated using permutation tests. The t4f (Tax4Fun) function in the R package of the metagenomics [55] was used to explore functional traits and predict metabolic capabilities based on 16S rRNA sequencing data and the KEGG pathway database. Mean relative abundance of prokaryotic taxa were calculated by dividing the number of sequences of that taxa by the total number of sequences in a sample. We used either t-tests for pairwise comparisons or ANOVA (using Type III Sums of Squares for Argentina samples) to examine differences in mean relative abundance of certain taxa between sites. Log transformations of the data were carried out when assumptions of normality or homogeneity of variances were not met.

## 3. Results

Soil analysis revealed few differences between urban, suburban, and rural sites in Buenos Aires, and only soil organic matter (%SOM) increased from rural to urban (Table 1). In Washington D.C., pH increased from rural to urban sites, while %SOM decreased along the gradient. Prokaryotic communities in each area were correlated to different soil parameters. In Buenos Aires, %SOM and %clay were significantly correlated to community composition (R^2^ = 0.63, *p* = 0.001 and R^2^ = 0.41, *p* = 0.014, respectively), and those variables were correlated to each other (r = 0.78). The Washington D.C. soil variables associated with prokaryotic communities were pH and soil organic matter (R^2^ = 0.58, *p* = 0.001 and R^2^ = 0.48, *p* = 0.009, respectively), and these variables were also correlated to each other (r = −0.75).

**Figure 2:**
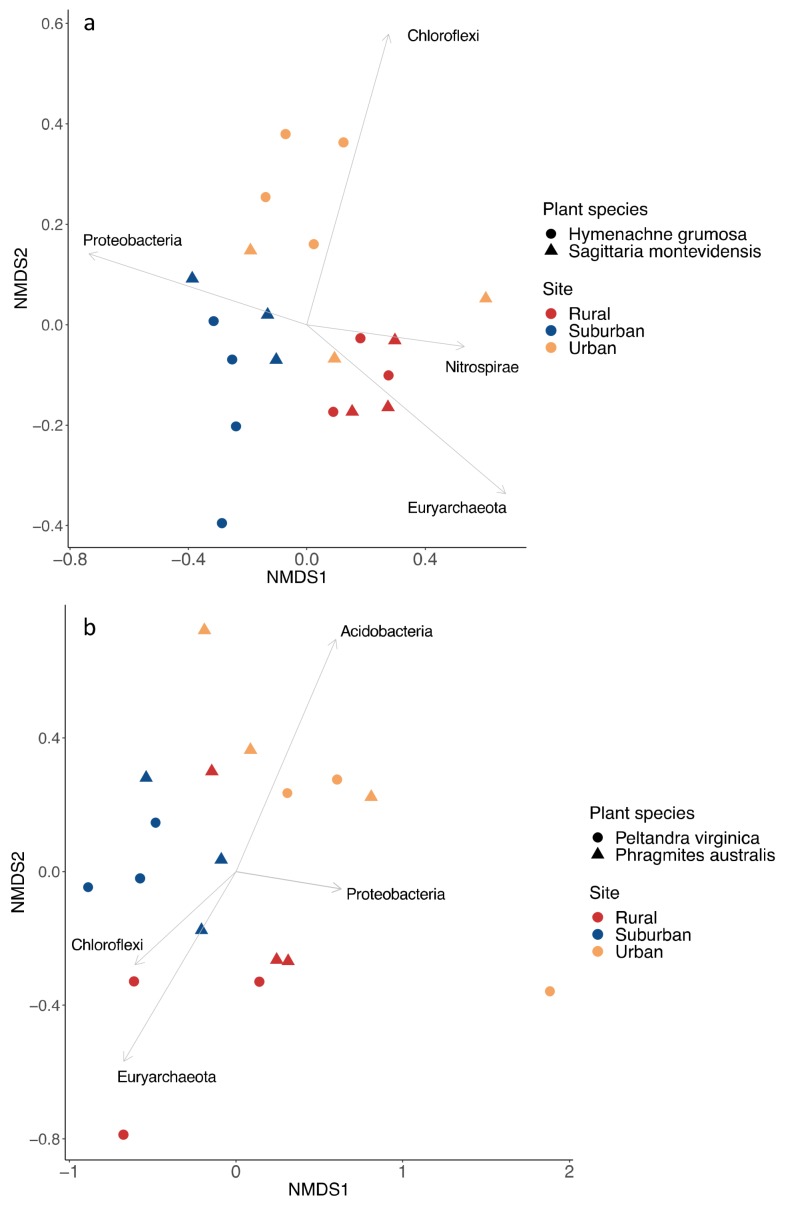
Non-metric multidimensional scaling ordination (NMDS) constructed using a Bray-Curtis dissimilarity matrix. Each point represents a sample with colors corresponding to sites, and shapes to plant species. The final stress values were 0.193 and 0.124 for the Buenos Aires (**a**) and Washington D.C. (**b**) ordinations, respectively. Vectors show the correlation of the most abundant phyla to community composition at the different sites.

Illumina sequencing generated approximately 3.7 million high quality sequences with a median of 76,000 sequences per sample (min = 11,811, max = 910,766). Nested PerMANOVA revealed that prokaryotic communities were significantly different along the urban gradient in Buenos Aires and in Washington D.C. (Buenos Aires: F = 2.6, *p* = 0.005; Washington D.C.: F = 2.3, *p* = 0.001), and differed between plant types at the *p* < 0.1 significance level (Buenos Aires: F = 1.5, *p* = 0.089; Washington D.C.: F = 1.3, *p* = 0.085) (Figure 2). Pairwise comparisons of the different sites showed that in Buenos Aires only urban and rural sites differed in community composition (*p* = 0.006), while in Washington D.C. the urban site communities differed from both the suburban and rural sites (*p* = 0.018 and *p* = 0.015 respectively). Shannon and Simpson diversity indexes did not differ between sites at either location (results not shown). The average ASV richness in urban, suburban, and rural sites in Buenos Aires was 751, 220, and 503, respectively. In Washington D.C., 2382 taxa were identified for the urban, 2347 for the suburban, and 2379 for the rural site.

The most abundant bacterial phyla were *Proteobacteria* (63.9%), *Firmicutes* (7.8%), and *Chloroflexi* (7.4%) in Buenos Aires; and *Proteobacteria* (16.5%), *Chloroflexi* (13%), and *Acidobacteria* (11.5%) in Washington D.C. The most abundant archaea phylum was *Euryarchaeota* in both areas (1.8% in Buenos Aires; 9.3% in Washington D.C.). In Buenos Aires, *Chloroflexi* was strongly correlated to urban communities, *Proteobacteria* to urban and suburban, and *Euryarchaeota* to communities in rural samples (Figure 2a). In Washington D.C., *Proteobacteria* and *Acidobacteria* were correlated to urban prokaryotic communities, while *Chloroflexi* and *Euryarchaeota* were better correlated to rural communities (Figure 2b). In both areas the most abundant class within *Euryarchaeota* was *Methanomicrobia* (56.5% in Buenos Aires; 44.9% in Washington D.C.).

**Figure 3:**
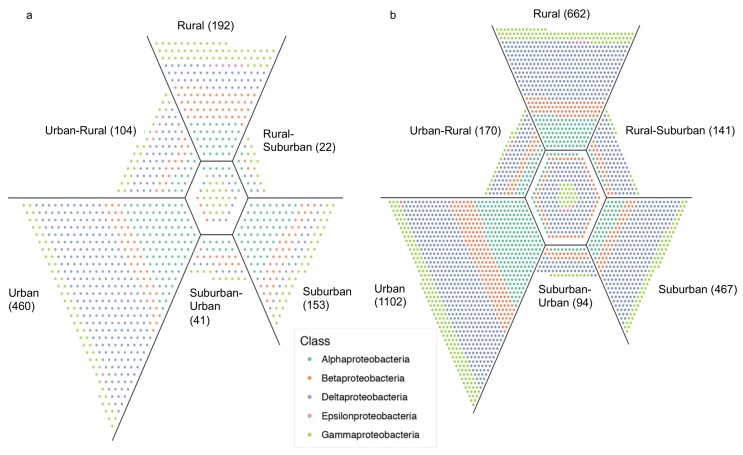
Venn-like representation of overlap between ASVs at urban, suburban, and rural sites for the phylum Proteobacteria. Each point represents an individual ASV with colors corresponding to different classes of Proteobacteria. Numbers in parenthesis indicate the total number of unique ASVs at each site. The center hexagon contains ASVs shared by all sites, while the trapezoids contain ASVs either exclusive to each site or those shared between two of them. In Buenos Aires (a) there were 45 ASVs shared by all sites and in Washington D.C. (b) 280. The plots were created using the unionplot R package [56].

Both urban sites had more unique *Proteobacteria* ASVs than suburban or rural sites (460 in Buenos Aires and 1102 in Washington D.C.) (Figure 3). Most unique ASVs in those urban sites belonged to the Class *Deltaproteobacteria*, with 201 unique ASVs in Buenos Aires and 493 in Washington D.C. This was particularly surprising in Buenos Aires, where relative abundance of *Deltaproteobacteria* did not differ between sites (F = 3.03, *p* = 0.075), while *Gammaproteobacteria* significantly increased from rural to urban sites (t = −2.26, *p* = 0.047), and comprised 93.4% of all Proteobacteria ASVs in the urban site. 

Within the *Gammaproteobacteria*, *Enterobacteriaceae* were most abundant in Buenos Aires and significantly higher in the urban site compared to the rural site (t = 3.6, *p* = 0.005). Bacteria putatively identified in the genus *Escherichia/Shigella* were exclusively found within the urban site. There was markedly higher relative abundance of coliform bacterial families in the suburban and urban sites compared with the rural site, which was mostly driven by *Enterobacter* and *Citrobacter* (Figure S2a in supplementary materials).

In Washington D.C., *Xanthomonadales* was the most abundant taxon within the *Gammaproteobacteria* and also showed a higher relative abundance in the urban than rural site (t = 2.58, *p* = 0.028). Members of the *Xanthomonadales* have the ability to break down PAHs, as well as other complex substrates [57,58]. Following this observation, other bacterial groups that have that capability were further explored. The relative abundance of members of the *Xanthomonadales* order (*Arenimonas*, *Dyella*, *Luteimonas*, *Lysobacter*, *Rhodanobacter*, and *Xanthomonas*), as well as other bacterial genera known to break down PAHs [59], were significantly higher in the urban sites relative to the suburban and rural sites (F = 4.4, *p* = 0.013) (Figure S2b in supplementary materials). 

**Figure 4:**
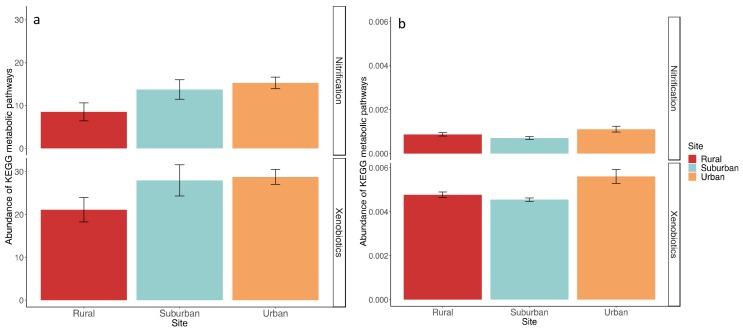
Bar plot showing mean abundance and standard error of metabolic pathways of nitrification and xenobiotic biodegradation across sites in Buenos Aires (**a**) and Washington D.C. (**b**).

KEGG metabolic pathways of the prokaryotic communities, and specifically those associated to xenobiotic biodegradation and nitrification, were investigated (Table S1 in supplementary materials). For the Buenos Aires samples, most ASVs could be mapped to KEGG organisms, allowing a relatively good functional prediction based on 16S rRNA data. The average FTU, which indicates the fraction of ASVs that could not be mapped to KEGG organisms, was 55% for rural, 36% for suburban, and 31% for urban sites. The obtained KEGG profiles suggest that functional traits were more similar between urban and suburban sites, while rural communities appear to be more functionally distinct (Figure S3a in supplementary materials). Metabolic pathways associated with expected urban stressors, such as pollution and excess nitrogen, were further analyzed. There was a significant enrichment in xenobiotic degradation pathways in the urban site relative to the rural site (t = 2.29, *p* = 0.05) and the same pattern was observed for nitrification pathways (t = 2.7, *p* = 0.025) (Figure 4a). In Washington DC, sites exhibited distinct metabolic capabilities (PerMANOVA F = 4.17, *p* = 0.007) (Figure S3b in supplementary materials), and an enrichment of xenobiotic degradation pathways in the urban relative to the rural site (t = 2.4, *p* = 0.049) was observed. Nitrification capacity was lowest in the suburban site and similar in the rural and urban sites (F = 4.52, *p* = 0.029) (Figure 4b). However, the average FTUs for the prokaryotic communities in Washington D.C. was much higher (around 92.5% on average), so only a small portion of the total community was represented in the KEGG profiles. Therefore, prediction of functional traits from taxonomic data in Washington D.C. would be inaccurate.

**Figure 5:**
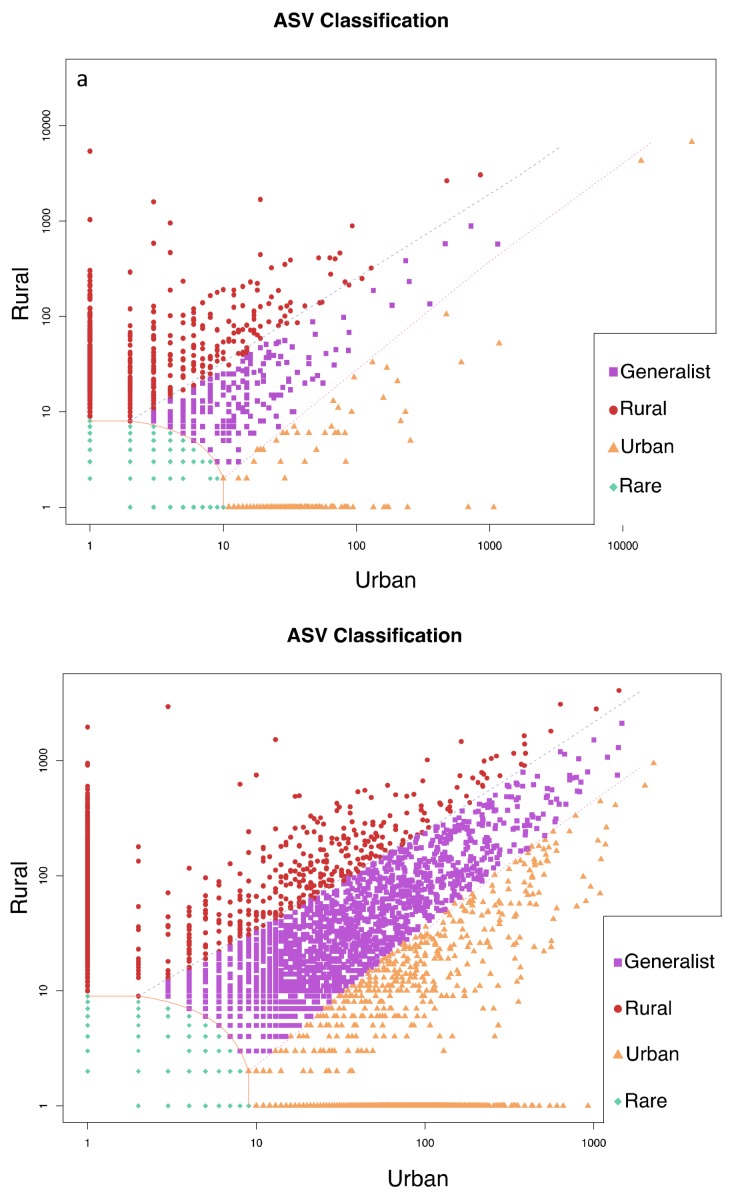
CLAM test results showing the classification of ASVs as habitat specialists or generalists based on relative abundance of species at each site for Buenos Aires (**a**) and Washington D.C. (**b**). ASVs were divided into four categories: urban specialists (Urban), rural specialists (Rural), generalists with no habitat preference, and too rare to classify (Rare). Note: some points are stacked so that specific values for each category can be found in the text.

Urban and rural sites in Buenos Aires and Washington D.C. contained ASVs that were classified as specialists for each habitat based on the CLAM test (Figure 5). In Buenos Aires, 5% were classified as urban specialists and 9% as rural specialists, while 82% of ASVs were too rare to classify. In Washington D.C., 21% were urban specialists, 16% were rural specialists, and 52% were too rare to classify.

In Buenos Aires only 12 taxa were indicators of the urban site while 182 were identified in the rural site. In Washington D.C. there were 153 indicator taxa of the urban and 130 of the rural site. Among these indicator taxa, those that had the highest fidelity and specificity values as defined by Dufrêne and Legendre [60] were further examined to identify useful indicator taxa and compared between Buenos Aires. This resulted in 6 urban and 46 rural indicator taxa in Buenos Aires and 26 urban and 37 rural indicators in Washington D.C. (Table S2 in supplementary materials).

Nitrite-oxidizing bacteria of the genus *Nitrospira* and *Nitrolancea* were indicators of the urban environments in Washington D.C. and Buenos Aires, respectively. Bacteria of the family *Nitrospiraceae* were also relevant indicators of the rural environments at both sites. In Buenos Aires, ammonia-oxidizing archaea within the *Thaumarchaeota* were indicators of the urban location and had significantly higher relative abundance in the urban site (F = 7.4, *p* = 0.006) (Figure S4 in supplementary materials). 

**Figure 6:**
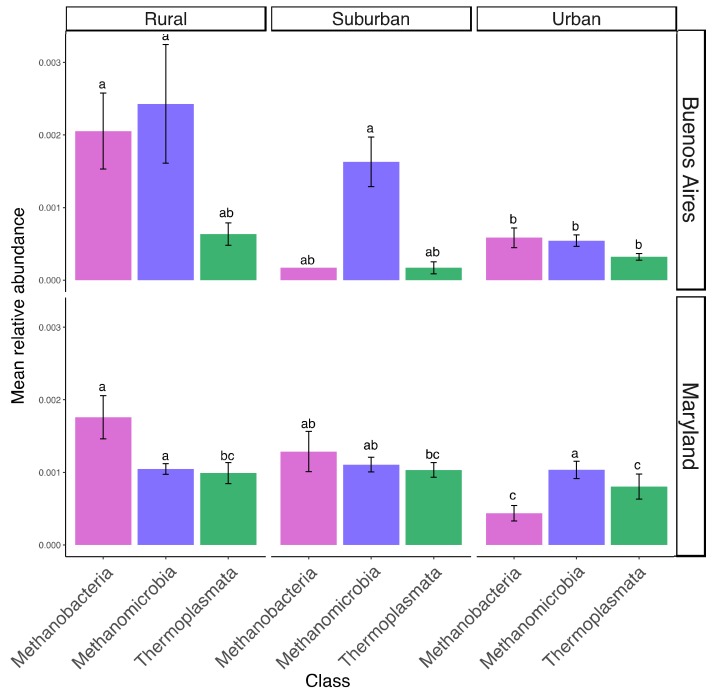
Mean relative abundance of Euryarchaeota classes for Buenos Aires and Washington D.C. in urban, suburban, and rural sites. Bars represent standard errors and different letters indicate significant differences between means (Tukey *p* < 0.05). Unclassified archaea are not included and only comprised 0.2% of total Euryarchaeota.

In addition to being highly correlated to rural sites in Buenos Aires and Maryland (Figure 2), *Methanobacteria* within the phylum *Euryarchaeota* were identified as rural indicators in both regions. Relative abundance of this phylum was significantly lower in urban than rural sites (Buenos Aires: t = 2.47, *p* = 0.03; Washington D.C.: t = 2.53, *p* = 0.03) and was mostly driven by a decrease in *Methanobacteriales* in both areas (Buenos Aires: t= 2.35, *p* = 0.04; Washington D.C.: t = 5.9, *p* < 0.001) (Figure 6). 

## 4. Discussion

In support of hypothesis one, we observed differences in the prokaryotic community composition across the urban to rural gradient (Figure 2). A common factor that shapes prokaryotic community structure is soil pH [18,61]. In our study, pH did not vary significantly across the urbanization gradient in Buenos Aires, but did increase with urbanization in Washington D.C. Additionally, pH was negatively correlated to %SOM. In contrast, SOM was higher in urban than rural sites in Buenos Aires. In both cases, %SOM was significantly correlated to community composition along the gradient. A study by Arroyo et al. [62] found similar results in natural and constructed wetlands, where SOM and not pH was the main soil variable related to microbial communities.

Species richness and diversity did not change significantly across the urban gradient in either location. Our results differ from those of other studies that found urbanization was negatively related to species richness and diversity in urban rivers [63,64], but agrees with one study on headwater streams, where alpha diversity did not change due to urbanization [65]. Even though we did observe enrichment of certain bacterial groups in the urban sites (Figure S2 in supplementary materials), richness and diversity indexes did not differ across the established gradient at either area. 

Consistent with previous studies in freshwater systems, *Proteobacteria* were the most abundant taxa in all sites [66,67]. This phylum was correlated with urban prokaryotic communities in Buenos Aires and Washington D.C. (Figure 2), and the Class *Gammaproteobacteria* was the main driver of that relationship in both cities. Associations between *Proteobacteria* and urbanization have been reported in other systems [63,68,69], and urban sites in our study had the greatest number of unique ASV’s (Figure 3). The nature of this association has been related to nutrient enrichment [63,70], which would be common in areas receiving sewage inputs or stormwater runoff. 

Within the Class *Gammaproteobacteria*, the order *Enterobacteriales* was the most prevalent across sites in Buenos Aires, while the order *Xanthomonadales* was the most abundant in Washington D.C. These orders were significantly more prevalent in urban rather than rural sites, suggesting that this could be related to specific environmental stressors at each of the urban sites. For example, some members of the *Enterobacteriales* are widely used as indicators of fecal contamination [71], and the presence of *E. coli* and other gastrointestinal associated bacteria in the urban site in Buenos Aires (Figure S2a in supplementary materials) was likely related to sewage flow in that area [34]. In addition to being common residents of the human intestinal tract, *Enterobacter* and *Citrobacter* are capable of degrading various types of hydrocarbons [72,73]. The increase of these genera in suburban and urban sites (Figure S2a in supplementary materials) may be attributed to heavy boat traffic and industrialization. This observation was supported by the community’s KEGG functional profiles that showed an enrichment in xenobiotic biodegradation pathways in the urban site (Figure 4a). 

In the Washington D.C. urban site, contamination with PAHs of industrial origin is a major environmental concern [74,75]. Although our study did not specifically test for PAHs, other studies observed an increase in these compounds in our specific urban location. Studies by Pinkney et al. [76,77] found that sediment PAH concentrations were considerably higher in the Anacostia than in the Choptank River (15–39mg/kg and 1.5 mg/kg dry weight respectively), where our urban and rural samples were collected. In our study, we found that there was a marked increase in the relative abundance of bacterial genera capable of degrading PAHs at the urban location relative to the rural and suburban sites (Figure S2b in supplementary materials). Our KEGG functional profiles were very limited at these sites, but the small subset of data obtained suggested that pathways associated to xenobiotic metabolism were more prevalent in the urban site, and these sites were functionally distinct from the rural sites (Figure 4b). We speculate that specific phyla were enriched in the urban sites in Buenos Aires and Washington D.C. as a result of major environmental pollutants affecting those areas, but functional gene analyses in future studies would help to corroborate this conclusion. 

Archaea of the class *Methanobacteria* were relevant indicators in rural sites of Buenos Aires and Maryland, and were significantly more abundant in soils from rural compared to urban locations (Figure 6). Nutrient additions have been related to decreases in methanogenesis [78,79], and nitrate concentrations were found to be relevant in structuring archaeal communities in an urban river [63]. Our urban sites did not have significantly higher levels of total nitrogen, but inputs of mineral nitrogen associated with more impervious surfaces and sewage inputs could explain the observed reduction in *Methanobacteria*. This can be confirmed for our Washington D.C. sites, where levels of ammonia were significantly higher in our urban compared to our rural site [22,24]. Even though we lack mineral nitrogen data for the Buenos Aires sites, metabolic pathways associated with nitrification were more prevalent in the urban than rural sites (Figure 4a), and ammonia oxidizing archaea that are stimulated by high organic nitrogen loads [80] were identified as relevant indicators of the urban site in Buenos Aires. In addition, we observed a significant increase in ammonia-oxidizing archaea and bacteria in the urban sites relative to the suburban or rural sites in Buenos Aires and Washington D.C. (Figure S3 in supplementary materials). These results suggest that excess nitrogen inputs are likely related to changes in prokaryotic community composition in our urban sites.

The largest number of unique ASVs corresponded to the *Deltaproteobacteria* in Buenos Aires and Washington D.C. (Figure 3). *Deltaproteobacteria* are capable of anaerobic respiration of nitrogen and sulfur compounds and degradation of organic compounds in wetlands [67]. This group includes multiple families of sulfate reducing bacteria, which can utilize a variety of C sources, including lipids and PAHs, to carry out dissimilatory sulfate reduction [81,82,83]. Some sulfate reducing bacteria can remove toxic materials from the water [84], and increases in these bacterial groups have been related to wastewater loadings in some freshwater wetlands [85]. In our urban sites, factors like stormwater runoff, hydrocarbon pollution, and sewage inputs could therefore explain the unique community of *Deltaproteobacteria*. 

*Chloroflexi* were significant components of the prokaryotic communities in both cities (Figure 2) and were predominantly represented by bacteria of the class *Anaerolineae*. A study on low tidal flats of an estuarine wetland found that the Class *Anaerolineae* was significantly correlated to total nitrogen and soil microbial respiration [86]. Members of the Class *Anaerolineae* are common components of anaerobic digesters, which would suggest that these bacteria could have an important role as part of the microbial heterotrophic community, but their ecological roles are still unknown [87]. Even though the functions of *Anaerolineae* remain elusive, they appear to be relevant in soil community structure and establish interactions with other groups of bacteria [88,89]. 

*Acidobacteria* belonging to the Class Subgroup 6 were the main group associated with urban communities in Maryland (Figure 2b). Bacteria belonging to this group have a complete set of genes to carry out assimilatory nitrate reduction and contain operons for detoxification of heavy metals [90]. Members of Subgroup 6 are abundant in nutrient rich soils and multiple groups of *Acidobacteria* can tolerate pollutants, such as PCBs and petroleum compounds. This has led to speculation that *Acidobacteria* might play a role in the degradation of such compounds [91]. 

Our results suggest that urban sites in Washington D.C. and Buenos Aires are subject to similar stressors, particularly higher concentrations of pollutants, and the shifts in prokaryotic community composition and putative functions reflected these conditions. Sewage inputs are also a concern at both urban sites and the presence of fecal bacteria in the Buenos Aires site was expected, as there is no sewage infrastructure. Our urban site in Washington D.C. also experiences frequent contamination from sewage [92], but our sampling time likely corresponded to a time when sewage concentrations were low. The closest combined sewer overflow outfall is located downstream of our sampling location and there were no heavy rainfall around our sampling date that would have resulted in overflow and subsequent upstream transport due to tides.

Our second and third hypotheses were not fully supported. Concerning hypothesis 2, which stated that prokaryotic communities would differ between plant species, we found that plant identity had a relatively small influence on community composition at each site (Figure 2). Therefore, factors associated with site differences had a greater role in structuring prokaryotic communities than plant properties. It is likely that other factors related to urbanization, such as various pollutants, override plant differences. Other studies in freshwater wetlands have found similar results, and concluded that either edaphic factors [93], site-specific factors [24], or certain landscape factors [94] have a greater effect in structuring prokaryotic communities. 

Concerning our third hypothesis, we did not find a specific indicator taxa that was common for both urban locations. This is in part due to the large difference between prokaryotic communities in the two regions. When we compared the two regions, they were more different from each other than across the gradients (data not shown). We did, however, observe that methanogens were relevant indicators of both rural locations, and identified other functionally similar indicators at the different sites. Nitrite-oxidizing bacteria were indicators of urban and rural areas in Washington D.C., and those belonging to the *Nitrospira* have been previously identified as common indicators of freshwater sediments [16]. Some organisms within the genus *Nitrospira* can carry out complete nitrification [95], which would make them a relevant functional group in urban as well as in rural sites, where agriculture is the prevalent land use. In Buenos Aires, *Chloroflexi* bacteria belonging to the genus *Nitrolancea* were indicators of the urban site. These nitrite-oxidizing bacteria have only recently been described, and are considered to be better competitors at higher levels of nitrite than *Nitrospira* [96]. The identification of similar functions among the different indicator taxa at each location suggests that studies of functional traits rather than specific taxa would be a better approach to characterize prokaryotic communities in these urban wetlands. 

The results of this study should be interpreted in light of the following limitations: we were unable to collect contaminant data for the different sites which could have helped support our findings, and functional data had to be inferred from community composition. In addition, sample size was low, and even though it was enough to detect site differences, a larger sample size might have allowed a better resolution of community differences between plant types. Studies of the proposed patterns and processes in other urban wetlands would be of interest.

## 5. Conclusions

Prokaryotic community composition shifted along the urban gradient in TFWs in Buenos Aires and Washington D.C. Given the important roles of bacteria and archaea in biogeochemical cycles, changes in community composition in these systems could have an effect on ecosystem function. In our study, differences in prokaryotic groups between sites likely reflected variation in environmental stressors, such as nutrient and hydrocarbon pollution. A loss of methanogens and an increase in nitrifying bacteria across the rural to urban gradient at both locations could have implications for nutrient and carbon processing in these systems, and might serve as an indicator of an altered state. Future studies of prokaryotic communities in other cities that experience either similar or different stressors would help confirm our observations. Our results also suggest that prokaryotic communities in these urban wetlands could be carrying out different functions than those in rural sites, particularly concerning pollutant transformation or removal. 

## Figures and Tables

**Table 1:** Soil characteristics for the urban, suburban, and rural sites within each area. Results are arithmetic means ± standard errors, except for texture results that are shown as percent values per site.

Parameter	Buenos Aires			Maryland		
	Rural	Suburban	Urban	Rural	Suburban	Urban
pH	6.4 ± 0.2	5.4 ± 0.08	5.8 ± 0.1	4 ± 0.3	4.7 ± 0.1	5.7 ± 0.1
SOM (%)	11.6 ± 0.5	6.6 ± 0.02	24.7 ± 2.2	35.6 ± 0.5	11.8 ± 1.2	6.5 ± 0.7
C (%)	2.7 ± 0.2	1.7 ± 0.1	3.54 ± 0.7	19 ± 0.2	5.6 ± 0.6	3 ± 0.4
N (%)	0.2 ± 0.02	0.2 ± 0.01	0.32 ± 0.1	0.12 ± 0.1	0.4 ± 0.1	0.2 ± 0.03
C/N	13	11.67	11.77	15.63	14.1	15.7
Sand (%)	0.31	0.38	0.33	0.26	0.35	0.17
Silt (%)	0.43	0.4	0.42	0.34	0.53	0.58
Clay (%)	0.25	0.22	0.25	0.4	0.12	0.26

## Supplementary Materials

The following are available online at https://www.mdpi.com/2076-2607/7/3/72/s1. Figure S1: Rarefaction curves. Figure S2: Enterobacteriaceae and PAH degrading bacteria. Figure S2: NMDS of KEGG metabolic profiles. Figure S3: Relative abundance of AOA, AOB, and NOB. Table S1: Functional traits. Table S2: Indicator species.
