# Supplementary material for: Urbanization Altered Bacterial and Archaeal Composition in Tidal Freshwater Wetlands Near Washington DC, USA, and Buenos Aires, Argentina
Source: Microorganisms. 2019 Mar 6;7(3):72. doi: 10.3390/microorganisms7030072 (PMC6463075; doi:10.3390/microorganisms7030072)
Supplement: Supplementary file 1 [file microorganisms-07-00072-s001.zip › Figure S1.pdf]

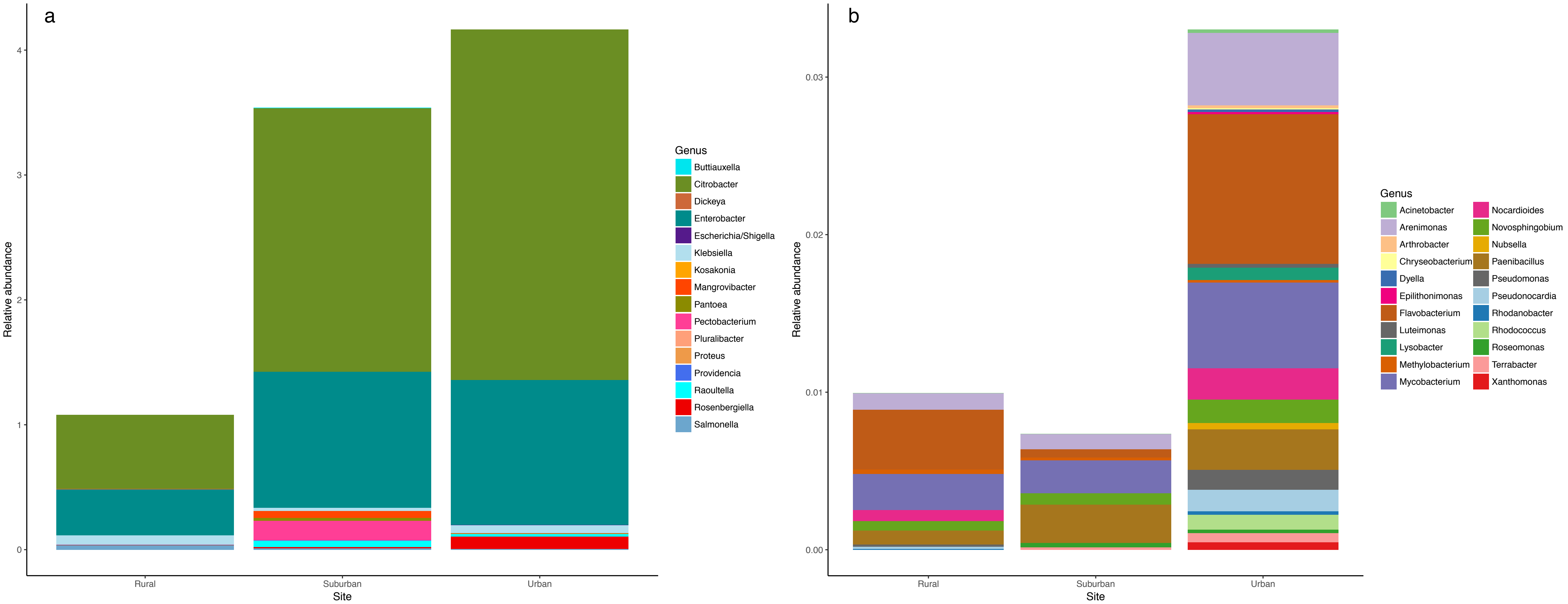

Figure S1: Relative abundance of bacteria of the family Enterobacteriaceae in Buenos Aires (**a**) and bacterial genus capable of PAH degradation in Washington D.C. (**b**)
