# Supplementary material for: Urbanization Altered Bacterial and Archaeal Composition in Tidal Freshwater Wetlands Near Washington DC, USA, and Buenos Aires, Argentina
Source: Microorganisms. 2019 Mar 6;7(3):72. doi: 10.3390/microorganisms7030072 (PMC6463075; doi:10.3390/microorganisms7030072)
Supplement: Supplementary file 1 [file microorganisms-07-00072-s001.zip › Figure S2.pdf]

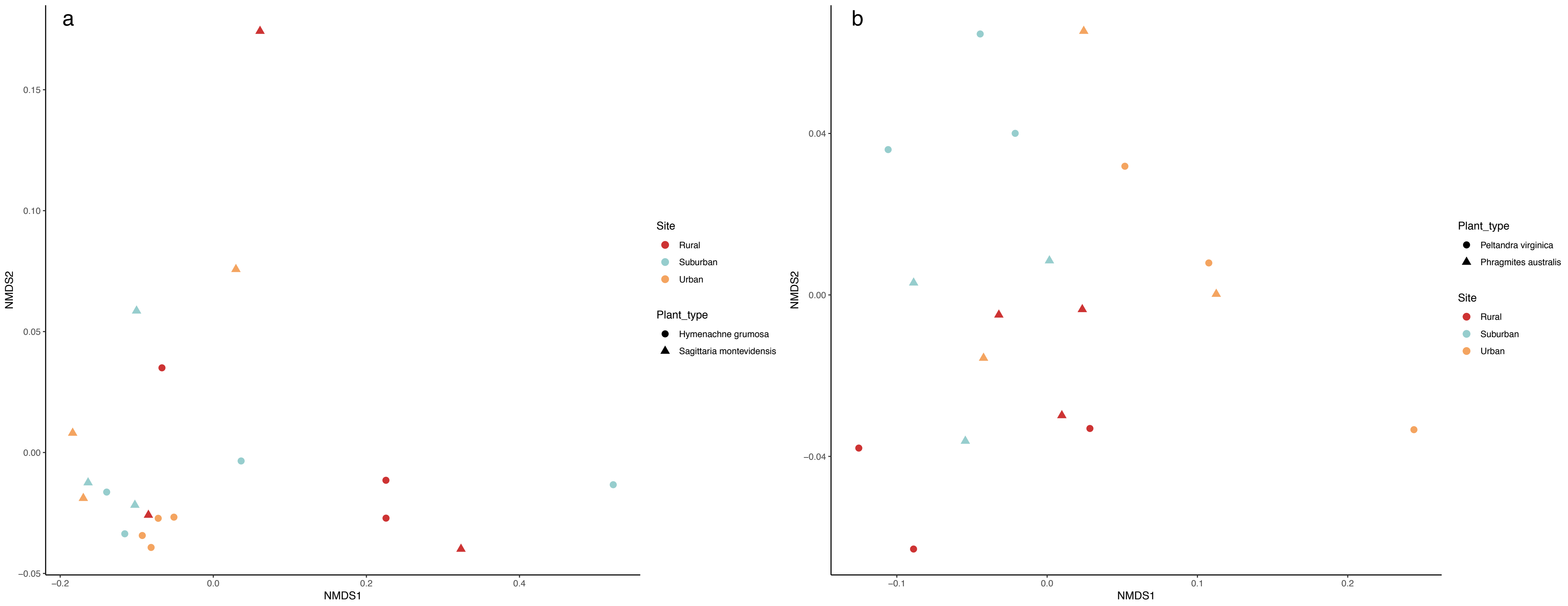

Figure S2: Non-metric multidimensional scaling ordination (NMDS) of KEGG metabolic profiles of the microbial communities. Each point represents a sample with colors corresponding to sites, and shapes to plant species.
