# Supplementary material for: Urbanization Altered Bacterial and Archaeal Composition in Tidal Freshwater Wetlands Near Washington DC, USA, and Buenos Aires, Argentina
Source: Microorganisms. 2019 Mar 6;7(3):72. doi: 10.3390/microorganisms7030072 (PMC6463075; doi:10.3390/microorganisms7030072)
Supplement: Supplementary file 1 [file microorganisms-07-00072-s001.zip › Figure S3.pdf]

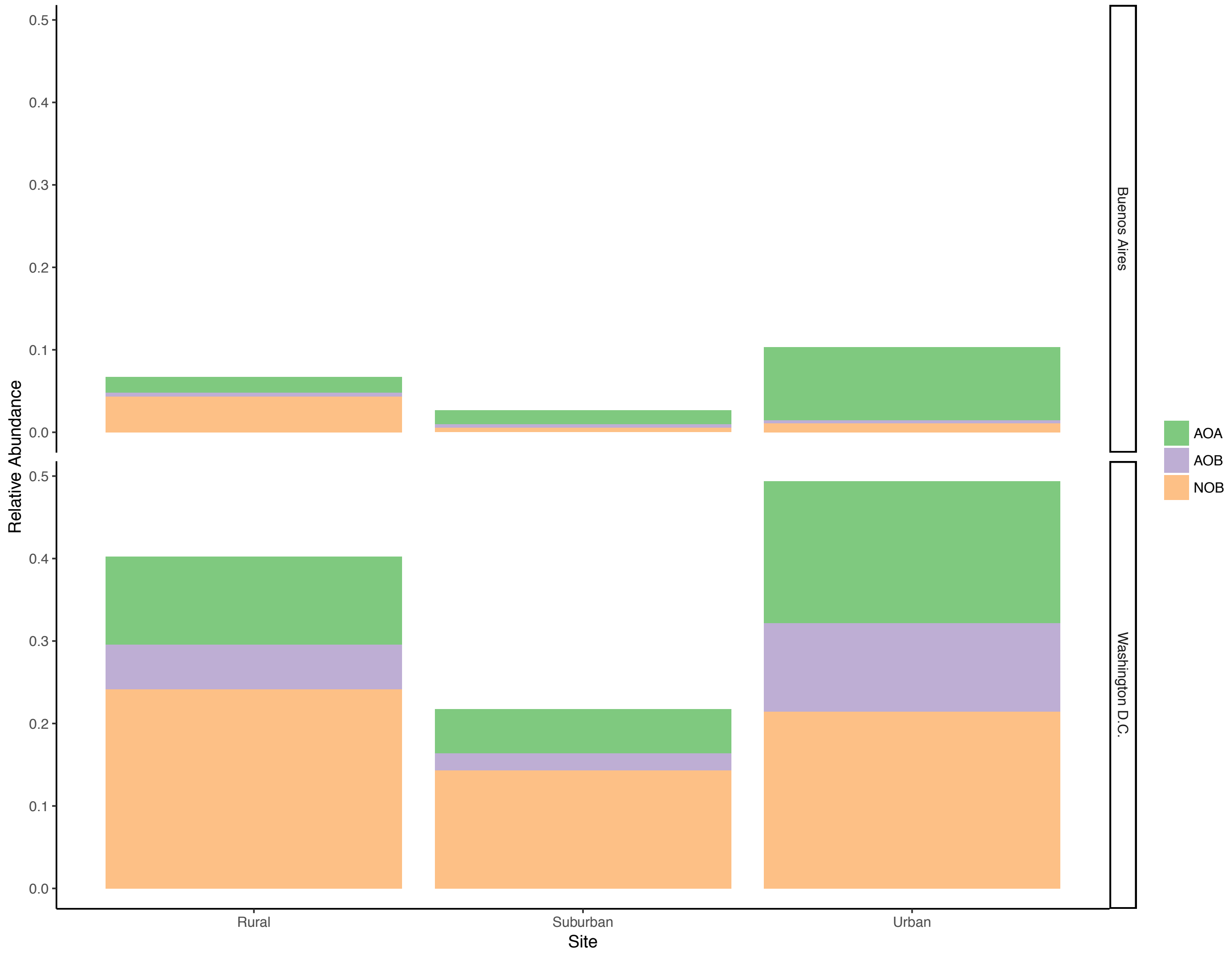

Figure 3: Relative abundance of ammonia-oxidizing archaea (AOA), ammonia-oxidizing bacteria (AOB) and nitrite oxidizing bacteria (NOB) along the urban gradient in Buenos Aires and Washington D.C.
